# Reactions among Indoor Pollutants

**DOI:** 10.1100/tsw.2001.75

**Published:** 2001-09-13

**Authors:** Charles J. Weschler

**Affiliations:** Department of Environmental and Community Medicine, University of Medicine and Dentistry/Robert Wood Johnson Medical School/Rutgers, 170 Frelinghuysen Rd., Piscataway, NJ 08854, USA

**Keywords:** indoor chemistry, ozone, hydroxyl radicals, fine particles, emissions, modeling

## Abstract

This paper reviews recent studies in the field of “indoor chemistry” — reactions among indoor pollutants. Advances have occurred in a number of areas. A mouse bioassay procedure has shown that ozone/terpene reactions produce products that are more irritating than their precursors, although the agents responsible for the deleterious effects remain to be determined. Indoor ozone/terpene reactions have been demonstrated to produce hydroxyl radicals, hydrogen peroxide, sub-micron particles, and ultrafine particles. New analytical techniques such as LC/MS and thermal desorption mass spectrometry have greatly improved our knowledge of the condensed-phase species associated with such particles. Indeed, the latter approach has identified a number of short-lived or thermally labile species, including organic hydroperoxides, peroxy-hemiacetals, and secondary ozonides, which would be missed by more conventional techniques. Investigators are making inroads into the poorly understood area of indoor heterogeneous chemistry. Systems studied include ozone/HVAC components, ozone/paint, and ozone/carpets. Another heterogeneous process that has been further examined is the indoor formation of nitrous acid through NO_2_ /surface chemistry. Emissions from indoor sources that contribute to, or are altered by, indoor chemistry have also received attention. Researchers have expanded our awareness of reactive chemicals that can emanate from wood coatings and other products commonly used indoors. In a related vein, a number of recent investigations have shown that emissions from materials can be significantly altered by indoor chemistry. On the theoretical side, an outdoor atmospheric chemistry model has been modified for use as an indoor air model, the effects of ventilation rates on indoor chemistry have been simulated, and initial steps have been taken in applying computational fluid dynamics (CFD) methods to indoor chemistry.

## INTRODUCTION

Almost 5 years ago we examined reactions and reaction products that could influence indoor environments[1]. In the intervening period there have been significant advances in "Indoor Chemistry". This paper will attempt to outline some of those advances. However, I shall begin by calling the reader's attention to Chapter 15, "Indoor air pollution: sources, levels, chemistry and fates", in the recent book by Finlayson-Pitts and Pitts titled *Chemistry of the Upper and Lower Atmosphere*[2]. The cited chapter contains an excellent summary of indoor chemistry through 1999. The fact that the topic of indoor chemistry has been included as a chapter in a textbook on atmospheric chemistry demonstrates that this field has reached a certain level of maturity. It is also a reminder that advances in outdoor atmospheric chemistry often provide insights relevant to indoor processes, despite the important differences between outdoor and indoor settings. (These differences include sunlight/photochemistry, precipitation, extent of temperature fluctuations, surface-to-volume ratios, and "ventilation".) The late Dr. Joan Daisey, director of the Indoor Environment Department at Lawrence Berkeley National Labs, used to refer to "...the continuum of indoor and outdoor air" and remind indoor researchers that they should remain cognizant of the total environment.

## RECENT STUDIES

### Mouse Bioassay Experiments

In papers[3,4] published in 1999 and 2000, Wolkoff and coworkers have described the application of the ASTM mouse bioassay procedure to assess the irritancy of products resulting from the reaction between ozone ($O_3$) and selected terpenes. This bioassay uses the reduction in the respiration rate of a mouse as an indicator of a chemical's airway irritancy. The investigators' initial paper[3] focused on α-pinene. They exposed the mice to an airstream that had a starting concentration of 6 ppm ozone and 80 ppm α-pinene. The mixture was 22 s old when it reached the breathing zone of a mouse, and the total exposure lasted 30 min. They found that the reduction in breathing rate resulting from exposure to the mixture (30%) was significantly greater than that produced by exposure to only ozone, α-pinene, or formaldehyde at concentrations comparable to those in the experiments. The sum of the effects of these compounds also was insufficient to account for the observed effect of the mixture. The results indicated that one or more unidentified products of the ozone/α-pinene reaction were responsible for the observed effect.

In a subsequent study[4], Wolkoff and coworkers examined d-limonene and isoprene, in addition to α-pinene, as reactants with ozone. In each of the experiments, the terpene (or isoprene) was present in large excess compared with ozone. Sampling with Tenax TA, followed by GC/MS analysis, was used to identify some of the stable, readily chromatographed products. The mixtures of α-pinene/ozone, d-limonene/ozone, and isoprene/ozone produced reductions in respiratory rates of 30%, 33%, and 51%, respectively. Once again, the investigators found that neither the reactants nor the identified reaction products were sufficient to explain the irritancy of the mixtures. They concluded, "One or more strong airway irritant(s) of unknown structure(s) were formed". Whether the unknown agent exists in the gas phase or the condensed phase, whether it is a short-lived, highly reactive intermediate or a stable, oxidized compound that requires special derivatiziation for detection, remains to be determined.

Rohr and colleagues[5] at the Harvard School of Public Health have replicated and extended the results of Wolkoff and his colleagues in Denmark. They have investigated ozone reactions with the same set of terpenes examined by the Danish group — α-terpene, d-limonene, and isoprene. There were slight differences in experimental design, but basically similar reductions in respiration rates were observed. Other physiological parameters were also monitored. Results from these studies are scheduled to be published shortly. Rohr et al. also measured fine and ultrafine particles in the ozone/terpene mixtures that were used in the mouse bioassay experiments[6]. Significant increases in particle concentrations were observed (see below).

A major criticism with each of these studies is the extremely high concentrations of reactants that have been used. When ozone levels are elevated indoors, the concentration is typically in the range of 20 to 40 ppb[7], compared with the 4000 to 6000 ppb (4 to 6 ppm) starting concentrations used in these studies. However, approximately 95% of the ozone is calculated to have reacted by the time the mixture reached the breathing zone of the mice, resulting in ozone levels closer to 300 ppb at the point of exposure. Average indoor concentrations of d-limonene and α-pinene are in the range of 2 to 5 ppb[8], compared with 50 to 80 ppm (50,000 to 80,000 ppb) used for limonene and α-pinene and 500 ppm (500,000 ppb) used for isoprene. In fairness, these average terpene concentrations are much lower than levels that can be achieved when certain products are used indoors. Personal experience tells us that it is not uncommon to detect the scent of lemon or pine in certain indoor settings. The odor thresholds of α-pinene and d-limonene are in excess of 400 ppb[9], indicating concentrations at least this high when their odor is apparent. Furthermore, the authors argue that it is important to consider the inherent sensitivity difference between mice and men, and suggest a ratio of 33:1 for relative sensitivity. Future human exposure studies to terpene/ozone mixtures may address some of these concerns (assuming that studies can be designed in such a way that the health of the human subjects will not be compromised).

## Ozone/Terpene Reactions

In the past 4 years, a number of research groups have identified a variety of products produced by ozone/terpene reactions in indoor settings. Among the products identified have been hydroxyl radicals (OH), hydrogen peroxide, submicron particles, and selected condensed-phase constituents of these particles.

### *Hydroxyl Radical*

Using an indirect technique to obtain a time-integrated signal, Weschler and Shields detected and quantified the hydroxyl radical (OH) in a commercial building[10]. For the purpose of the measurements, the conditions in the office setting were manipulated, but they were representative of conditions that occur naturally. During monitoring periods when the concentrations of both ozone ($O_3$) and d-limonene were elevated and the air-exchange rate was moderate, they found the average indoor OH concentration to be approximately $7 \times 10^5$ molecule/cm$^3$. This value is lower than typical outdoor midday values (~$5 \times 10^6$ molecule/cm$^3$) but larger than outdoor nighttime values. The results confirmed findings from an earlier modeling paper[11] by the same authors indicating that reactions among $O_3$ and olefins can generate meaningful quantities of OH under conditions that commonly occur indoors. Subsequent reactions between the hydroxyl radical and other indoor pollutants can generate still other radicals, as well as stable oxidized compounds containing one or more –C=O (carbonyl), –COOH (carboxylate), and/or –OH (hydroxyl) functional groups. Some of the radicals that are subsequently generated (e.g., $HO_2$, $CH_3O_2$, and other organic peroxy radicals) may be more relevant to irritancy and health concerns that the hydroxyl radical itself.

## Particles

While conducting the experiments to measure indoor hydroxyl radicals generated by ozone/limonene reactions, the author and his colleague, H. Shields, noticed that the white board in the ozone/limonene office was turning slightly yellow. We speculated that the yellowing was due to soiling by particles (secondary organic aerosols) generated as a consequence of the ozone/limonene reaction. This is not unexpected. Reactions between ozone and unsaturated hydrocarbons produce both gas-phase and condensed-phase species. Grosjean et al.[12,13] have estimated that 20% of the products produced in the ozone/d-limonene reaction occur as aerosols, and smog-chamber studies have demonstrated that high aerosol yields result from the reaction of ozone with terpenes. Hence, we decided to investigate the formation of particles in a more systematic fashion[14]. In a set of experiments conducted in adjacent identical offices, ozone and a terpene (either d-limonene, α-terpinene or a commercial terpene-based cleaner containing α-pinene) were deliberately introduced into one office, while the other office served as a control. Subsequent particle increases and redistribution were monitored with an eight-channel laser particle counter. (Note that this instrument was unable to monitor particles smaller than 0.1-µm diameter.) For each terpene examined, particle concentrations were significantly larger in the office with the added ozone (200 to 300 ppb) than in the control office without the added ozone. This difference was greatest for particles in the 0.1- to 0.2-µm diameter size range; over time, differences developed in the larger size ranges. The particle increases were also greatest when the terpene was d-limonene, although significant particle increases were noted for each of the selected terpenes. In the case of d-limonene, the number concentration of particles in the 0.1- to 0.2-µm size range was as much as 20 times larger in the office with the added ozone than in the control office ($6 \times 10^3$ vs. $3 \times 10^2$ particle/cm$^3$). In another set of experiments, much closer to real world conditions, d-limonene was deliberately introduced into one of the offices, but ozone was **not** added to either office. Instead, the indoor ozone concentrations were those that happened to be present as a consequence of outdoor-to-indoor transport — typically between 2 and 40 ppb. In the office that contained supplemental d-limonene, the concentrations of the 0.1- to 0.2-µm particles tracked those of indoor ozone (the limiting reagent) and were as much as 10 times greater than levels measured in the comparable office that did not contain supplemental d-limonene. At an indoor ozone concentration of 23 to 28 ppb, the mass concentration difference (sub-micron particles) between the two offices was approximately 20 µg/m$^3$.

In a follow-up study Weschler and Shields[15] explored the influence of air-exchange rates on particle counts and the evolution of particle size distributions. The experimental setup was similar to that described above, employing adjacent, unoccupied offices. Both rooms contained equivalent limonene sources, but only one room contained an ozone generator. The evolution of the particle size distribution was monitored through several cycles in which the ozone generator was turned on, operated for a number of hours, and turned off. (The ozone generator was turned on and off remotely, without opening the office door and disturbing conditions in the room). The air-exchange rates during the experiments were either high (working hours) or low (non-working hours). Both the growth in particle counts and evolution of the size distribution were strong functions of the air-exchange rate. The results were consistent with semivolatile products of the ozone/limonene reactions condensing onto or partitioning into existing aerosols. At low air-exchange rates, the particle counts in the 0.1- to 0.2-µm diameter size range peaked at ~35,000 particles/cm$^3$; peaks also occurred in the next 3 size ranges, separated by 30-min intervals. At high air-exchange rates, the particle counts in the 0.1- to 0.2-µm diameter size range peaked at less than 1000 particles/cm$^3$; there was no discernable growth in the larger size ranges. In general, as the air-exchange rate decreased, particle size distributions were shifted toward larger particle diameters, and more time was required to reach a stable distribution.

Wainman et al.[16] used a nested, dynamic chamber to further examine the generation of secondary organic aerosol as a consequence of the reaction of ozone with d-limonene. The nested chamber design consisted of a smaller chamber (2.5 $m^3$) surrounded by a larger one (25 $m^3$), with air exchange occurring between the two. The inner chamber represented an indoor environment and was operated at an air-exchange rate below 1 $h^{-1}$, while the outer chamber represented an outdoor environment and was operated at an air-exchange rate of approximately 45 $h^{-1}$. Limonene was introduced into the inner chamber either by the evaporation of reagent grade d-limonene or by inserting a lemon-scented solid air freshener. Ozone was introduced to the inner chamber in a series of injections, with multiple injections occurring in a given experiment. Following an injection, the concentration of ozone was allowed to decay before another injection was made. Particle levels were monitored using an eight-channel optical particle counter. Of the size ranges being monitored, measurable particle formation and growth occurred almost exclusively in the 0.1- to 0.2-µm and 0.2- to 0.3-µm size fractions in all of the experiments. Particle counts increased in the 0.1- to 0.2-µm size range soon after ozone was introduced, but an increase in particles in the 0.2- to 0.3-µm size range did not occur until at least the second ozone injection. One of the solid air freshener experiments produced an estimated particle mass concentration in excess of 20 µg/$m^3$ as the result of four ozone injections made over a period of 10 h. The results of this study reaffirm the potential for significant increases in indoor submicron particle concentrations as a result of the formation of secondary organic aerosols via the ozone/limonene reaction.

Each of the previously outlined particulate studies only monitored particles larger than 0.1-µm diameter. This is an inherent limitation of the optical/laser particle counters that were utilized. Hence, no information was obtained regarding changes in particle number concentrations and particle size distributions for particles in the size range of approximately 10 to 100 nm (0.01 to 0.1 µm) — so called ultrafines. In preparation for inhalation-exposure experiments in mice (see above), Rohr et al., at the Harvard School of Public Health, have recently obtained information in this size régime[6]. The studies were conducted, in part, to better understand the effects of varying air-exchange rates on the size distribution of ultrafine particles to which the mice might be exposed. The experiments involved reactions between ozone and α-pinene occurring in a ventilated Plexiglas chamber. A Scanning Mobility Particle Sizer (SMPS) with a size range of 15 to 777 nm was used to determine the total number concentration and size distribution of the aerosol throughout the experiments. The concentrations of α-pinene and ozone were also monitored. The α-pinene concentration was held approximately constant at 80 to 109 ppm. Ten different ozone concentrations were used, ranging from 91 to 367 ppb. The chamber residence time, determined by the air-exchange rate, was 6.4 to 7.1 min. At "low" ozone levels (99 to 121 ppb), particle growth was observed in the chamber, with condensation and/or coagulation occurring to increase mean particle diameter. This effect was not observed at "high" ozone concentrations (313 to 367 ppb), where steady state was reached very rapidly and with little change in particle diameter. Preliminary findings indicated a positive nonlinear relationship between ozone concentration and total steady-state number concentration. The results enhance the understanding of the mouse bioassay experiments. However, the concentrations employed were quite high, and caution should be exercised in extrapolating the results of these experiments to typical indoor conditions with orders of magnitude smaller α-pinene concentrations.

Long et al.[17], in a contribution that is also from the Harvard School of Public Health, have examined the generation of ultrafine particles as a consequence of ozone/terpene chemistry under quite realistic conditions. The investigators monitored six cleaning events in residential indoor environments. The commonly available cleaning agent was a pine oil–based cleaner that contains a significant amount of α-pinene and lesser amounts of other terpenes and terpene alcohols. In five of the six events, there was significant generation of ultrafine particles. Three of the events involved mopping floors with a dilute cleaning solution; two events involved toilet cleaning. The outdoor ozone concentrations (1 to 52 ppb) during these events were monitored, as were the mean air-exchange rates. The ozone within the residences was present as a consequence of outdoor-to-indoor transport. No ozone-generating device was used. The investigators found that more than 50% of the particle increase (by volume) occurred in the ultrafine size range. They also found that number concentrations (peak values) increased from 7- to 100-fold as a consequence of these cleaning events and that mass concentrations for particles smaller than 2.5 μm increased as much as 32 $\mu g/m^3$. In one home, 10 min of mopping with the diluted cleaner influenced particle concentrations for more than 8 h. The paper presents detailed particle distribution profiles for this 8-h period. The peak in the particle number concentration shifts from about 60-nm particles 10 min after mopping to 110 nm 1 h after mopping, and to about 120 nm 2 h after mopping. In summary, this work shows that indoor chemistry can be a significant source of ultrafine particles during and after routine cleaning if ambient ozone is at even a modest concentration.

The above-cited studies do more than illustrate the potential for ozone/terpene reactions to contribute to the overall concentration of submicron particles in indoor air; they also demonstrate a coupling between outdoor ozone and indoor submicron particles. Since outdoor submicron particles correlate with outdoor ozone levels, indoor particles generated from ozone/terpene reactions can vary coincidentally with outdoor fine particles. This coupling is relevant when considering the epidemiological studies that demonstrate an association between incremental changes in outdoor particle concentrations and increases in the rates of morbidity and mortality.

## *Particulate Constituents*

In the past 5 years there have been a number of creative, carefully executed studies that have further characterized the products of ozone/alkene and, more specifically, ozone/terpene chemistry. Although these studies have not focused on indoor environments, their results are important as we try to understand the consequences of ozone driven chemistry in indoor settings.

Tobias and Ziemann[18] have used a thermal desorption particle beam mass spectrometer to investigate the organic aerosol formed when ozone reacts with 1-tetradecene in the presence of alcohols or carboxylic acids. The approach has identified two major types of aerosol products. The more volatile products are organic hydroperoxides formed by the reaction of alcohols or carboxylic acids with stabilized Criegee biradicals. The less volatile products are peroxy-hemiacetals, formed by reaction of the organic hydroperoxides with tridecanal, a primary product of the ozone/tetradecene reaction. In a companion paper[19], these authors and additional coworkers have examined the constituents of the aerosol formed in the ozone/tetradecene reaction under humid and dry conditions. Under humid conditions the major products are an organic hydroperoxide and a peroxyhemiacetal. Under dry conditions the major products are secondary ozonides. Significantly, when the constituents of the ozone/tetradecene-produced aerosols were analyzed by standard gas-chromatographic procedures, the organic hydroperoxides, peroxy-hemiacetals, and secondary ozonides were **not** detected. Instead, these compounds decomposed to more volatile species. Reactions analogous to those that occur in the ozone/tetradecene system are expected to occur in ozone/terpene systems. The resulting peroxides and secondary ozonides are potentially irritating to human occupants. However, their presence in indoor air would be missed by conventional analytical techniques.

Glasius et al.[20] identified a number of aerosol constituents produced by the reaction of ozone with selected terpenes. Six different cyclic monoterpenes were studied, including α-pinene and d-limonene. The analysis focused on carboxylic acids and used derivatization techniques followed by GC/MS analysis or, a newer approach, high pressure liquid chromatography (HPLC)/MS analysis. The identified acids included dicarboxylic acids, oxocarboxylic acids, and hydroxyketocarboxylic acids. Furthermore, the results indicate that water vapor may play a role in the formation of these aerosol constituents. Additional studies of the products of ozone/terpene (isoprene) reactions have been reported by Yu et al.[21,22], Jang and Kamens[23], and Virkkula et al.[24]. Each of these studies used special chemical derivatization techniques to render the identified products chromatographable. In some cases, the derivatization techniques facilitated the identification of multifunctional, highly oxidized compounds that had not been detectable by methods previously applied to these products. Taken together, the studies just described have greatly improved our knowledge of the condensed-phase products generated by ozone/terpene reactions. More specifically, higher molecular weight products expected when ozone reacts with d-limonene in indoor settings include limonic acid, limononic acid, 7-hydroxylimononic acid, 7-hydroxy-keto-limononic acid, 4-methyl-3-(3-oxobutyl)pent-4-enal, and 3-acetyl-6-oxoheptanal. Similarly, in the case of α-pinene, expected higher molecular weight products include pinic acid, norpinonic acid and isomers, hydroxy pinonic acid, hydroxy pinonaldehydes, and pinonic acid.

## Hydrogen Peroxide

In a series of experiments similar to those used to measure indoor hydroxyl radicals (i.e., conducted in a realistic but manipulated indoor environment) Li et al.[25] measured significant concentrations of hydrogen peroxide formed as a consequence of ozone/limonene reactions. The ozone levels used in these studies were between 80 and 175 ppb, while the d-limonene levels were between 100 and 360 ppb. The resulting $H_2O_2$ concentrations ranged from 0.58 to 1.5 ppb and varied with air-exchange rate as well as reactant concentrations. At moderate air-exchange rates (~1 to 2 $h^{-1}$), hydrogen peroxide concentrations were approximately a factor of two greater than at elevated air-exchange rates (~12 $h^{-1}$). In this study, input ozone concentrations were smaller than d-limonene concentrations, with the exception of two experiments. The yield of hydrogen peroxide, based on the limiting reactant, ranged from 0.6 to 1.9%, consistent with values reported in the literature. In addition to $H_2O_2$ formation, there was also significant production of submicron particles, as would be expected given the studies discussed earlier. The results show that surface removal is a much greater sink for $H_2O_2$ than for the submicron particles (a 1$^{st}$-order rate constant for surface removal of approximately 6 $h^{-1}$ vs. 0.2 $h^{-1}$). This work indicates that, under appropriate conditions, indoor hydrogen peroxide concentrations are comparable to outdoor values. Hydrogen peroxide is extremely water soluble; since the particles formed from ozone/terpene reactions are hygroscopic, a fraction of the co-occurring $H_2O_2$ is expected to partition into the particle phase. This is noteworthy because Friedlander and Yeh[26] have suggested that association with particles facilitates $H_2O_2$ transport to the lower lung, and particle-associated $H_2O_2$ is capable of contributing to tissue damage and other adverse effects if exposures are sufficient.

## Peroxyalkylnitrates

The following speculation is peripherally related to ozone/terpene chemistry. In our earlier paper[1] on reactions among indoor pollutants, we speculated that the highly irritating compound peroxybenzoyl nitrate might be formed indoors as a consequence of reactions between hydroxyl radicals and benzaldehyde in the presence of $NO_2$. We now realize the requisite conditions may occur when ozone, certain terpenes, styrene, and $NO_2$ are present simultaneously at low air-exchange rates. This mixture will yield both hydroxyl radicals and benzaldehyde, which can subsequently react as noted above. The simultaneous occurrence of such compounds in indoor settings is not unusual. Furthermore, analogous chemistry can produce other noxious peroxyalkylnitrates. Studies designed to detect such organic nitrates, under suitable indoor conditions, are warranted.

## Indoor Heterogeneous Reactions

### Ozone/HVAC Components

Mechanically ventilated buildings use a system of supply fans and ductwork to provide air to various compartments. If ozone is present in the circulated air, there is the potential for it to react with the surfaces of the heating, ventilating, and air conditioning (HVAC) system. Such reactions could be the source of pollutants in the supply air. Morrison and coworkers[27] have examined heterogeneous processes related to this concern. They collected samples of volatile organic compounds (VOCs) and aldehydes from the ventilation system of a new building. In general, they found that the contribution of these materials to VOCs and aldehydes in buildings are "… likely to be low". They also exposed components of HVAC systems to 100 ppb of ozone in 10.5-l chambers that were ventilated at 5.7 $h^{-1}$. The emission rates of aldehydes from duct liner, duct sealing caulk, and neoprene gaskets were larger after exposure to ozone. In particular, the concentrations of n-C5 to n-C10 aldehydes increased markedly. In the case of a new neoprene duct liner, they measured a combined n-C5 to n-C10 aldehyde emission rate of approximately 400 $\mu g/m^2$ h — large enough to impact the indoor concentrations of these aldehydes under typical conditions.

### Ozone/Paint

Paints, especially "low VOC paints", commonly use linseed oil as a drying agent. Linseed oil contains a number of unsaturated fatty acids, including linoleic acid and linolenic acid. Andersson et al.[28] have demonstrated that, when such paints are used indoors in the presence of ozone, a series of aldehydes and organic acids are produced. Major products include propanal, hexanal, propanoic acid, and hexanoic acid, with lesser amounts of formaldehyde, acetaldehyde, pentanal, octanal, and nonanal.

Among the parameters used to describe the interaction of ozone with surfaces is the "mass accommodation coefficient", which is also referred to as the "uptake coefficient" or "reaction probability". This unitless parameter can be viewed as the number of ozone "sticks" per total number of collisions, or the rate of ozone loss on a surface normalized by the rate of ozone-surface collisions. It varies with the nature of the surface, compounds adsorbed on the surface, temperature, and relative humidity. Typical[29] mass accommodation coefficients for ozone striking glass, latex paint, and wall paper (vinyl and paper) are in the range of $10^{-5}$ to $10^{-7}$. When an ozone molecule "sticks" to a surface, it can react with surface constituents to produce products, including VOCs, or decompose through a series of surface-assisted steps. For a given VOC, the *VOC formation factor*, $\kappa$, is defined as the number of molecules of VOC formed on the surface divided by the total number of "sticks". Different VOCs have different VOC formation factors. Once a VOC is formed on a surface, it can leave the surface and enter the gas phase. Reiss et al.[30] determined VOC formation factors for reactions between ozone and various types of interior latex paints. Four different brands of paint were applied to the inside surfaces of glass tubes that served as flow reactors. Air containing ozone (49 to 147 ppb) was passed through the tubes and the concentrations of selected organics were measured upstream and downstream of the tube. The flow of ozone through the tube was too fast for homogeneous chemistry to occur; the only reactions that were possible were heterogeneous reactions. The authors found that, for two of the paints, formaldehyde was produced in significant amounts — the formaldehyde formation factor for these paints ranged from 0.1 to 0.3. The authors hypothesized that the formaldehyde resulted from ozonation of unsaturated residues in the vinyl resins of the paints. Based on these results, they conducted modeling studies that indicated that heterogeneous reactions between ozone and certain latex paints could contribute significant quantities of formaldehyde to indoor settings.

## Ozone/Carpets

One of the early studies related to indoor chemistry involved ozone and carpet emissions[31]. Morrison and Nazaroff[32] have extended those early results. They aged four different samples of carpet in a ventilated enclosure for more than a year. The samples were then exposed to 100 ppb of ozone in a 10.5-l chamber for 2 to 5 days. Oxidized organic compounds coming off of the carpets were measured during and after the ozone exposure. One of the carpets — a residential, nylon fiber, cut pile carpet — emitted significant quantities of n-C4 to n-C11 aldehydes, as well as 2-nonenal. The 2-nonenal is especially significant since it has a very low odor threshold — 0.002 ppb. Even when the ozone exposure ceased, the carpets continued to release the aldehydes. These compounds apparently sorb to the carpet surface after they have formed, and the carpet becomes a reservoir for the aldehydes, releasing them long after the ozone exposure ends. Using a steady-state model and the measured emission rates, Morrison and Nazaroff estimated that "occupants could smell these compounds for years after installation of this carpet in a typical home". Morrison has further suggested that ozonide intermediates may help to explain the somewhat delayed release of certain oxidation products, such as 2-nonenal, from carpet surfaces[33]. This is an interesting hypothesis with broad implications and warrants further examination.

Morrison and Nazaroff[34] examined the rate at which ozone is removed by whole carpet, carpet fibers, and carpet backing. For whole carpet and carpet-backing samples, they measured mass accommodation coefficients on the order of $10^{-5}$ to $10^{-4}$ (based on the nominal surface area of the carpet, rather than its total surface area). These values are comparable to the values reported by Reiss et al.[29] for latex paints. For carpet fibers, they measured values on the order of $10^{-7}$ to $10^{-6}$ (based on estimated fiber area). The values measured for the mass accommodation coefficients decreased as the duration of a sample's ozone exposure increased.

## Nitrous Acid

In our earlier paper on indoor chemistry[1], we noted that the indoor generation of nitrous acid (HONO) is primarily a consequence of heterogeneous reactions involving nitrogen dioxide ($NO_2$) and water (see equations 17 and 18 in Reference 1). Indeed, to the author's knowledge, the investigations of this reaction by J. Pitts and colleagues[35,36] were among the first examples of indoor chemistry published in the literature. There have recently been a couple of studies that provide additional information on the relative significance of this reaction and the factors that influence it. Lee et al.[37] have used passive samplers to measure the concentrations of HONO and $NO_2$ in 119 Southern California houses. Indoor HONO levels averaged 4.6 ppb, compared to 0.8 ppb for HONO outdoors. As expected, there was a strong correlation between indoor HONO levels and indoor $NO_2$ levels; indoor HONO concentrations averaged 17% of indoor $NO_2$ concentrations. Several housing characteristics, including the use of air conditioning, humidification, and gas ranges, were examined for possible correlations with both pollutants. As expected, the indoor levels of $NO_2$ and HONO were positively associated with the presence of gas ranges. Interestingly, the investigators found that indoor HONO averaged 5.9 ppb in 58 homes with humidifiers and 2.6 ppb in 32 homes without humidifiers. No explanation was presented for this finding. However, a recent paper by Wainman et al.[38] bears directly on this observation.

Wainman et al.[38] used a nested-chamber design identical to that employed in their ozone/limonene studies (see above)[16] to examine the influence of surface type and relative humidity on the indoor generation of HONO. Three different surface types — Teflon, wallpaper, and carpet — and two relative humidities (RH) — 50 and 70% — were investigated. The results showed that, compared with Teflon surfaces, carpet made of synthetic fibers increased the $NO_2$ surface removal rate by nearly an order of magnitude and resulted in higher peak HONO concentrations. The results also indicated that the capacity of a surface to sorb water will determine if HONO is released from that surface after the heterogeneous reaction between $NO_2$ and sorbed water is no longer significant. At both 50 and 70% RH, vinyl-coated wallpaper was found to release HONO for prolonged periods of time after the $NO_2$ source was turned off. In contrast, Teflon was found to do so only at 70% RH (and not at 50%). These results build on the earlier findings of Spicer et al.[39]. One implication of these experiments is that indoor HONO levels are strongly influenced by both the relative humidity and the tendency of a surface to sorb moisture. This finding provides an explanation for the correlation between indoor HONO levels and humidification reported by Lee et al.[37] (see previous paragraph). The highest and most prolonged HONO concentrations are anticipated to occur during periods of elevated relative humidity in households that utilize unvented gas appliances and have surfaces that readily sorb water from the air. The fate of HONO in indoor air warrants further investigation.

## Emissions Contributing to, or Altered by, Indoor Chemistry

### *Wood Coatings*

In two recent papers[40,41], Salthammer has reported measurements of reactive compounds and secondary products from water-based and UV-cured coatings. The first paper[40] described a series of chamber studies in which emissions of coatings used on wood furniture were sampled and analyzed. The identified compounds included 4-methyl-2-pentanone (MIBK) and 3,5-dimethyl-1-hexyne-3-ol. Neither of these species was anticipated to be present. Subsequent investigation revealed that a wetting and defoaming agent identified as 2,4,7,9-tetramethyl-5-dicyne-4,7-diol (T4MDD) can decompose to yield the two compounds just mentioned. T4MDD is an additive in a number of water-based lacquer systems intended for use on wood furniture.

UV-curable coatings contain photoinitiators. Fragmentation processes involving these photoinitiators can generate radicals that can, in turn, contribute to indoor pollution[41]. An example of a compound formed in this fashion is 1-phenyl-2-hydroxy-2-methyl-propane-1-one (PHMP). This compound can participate in subsequent chemistry leading to still other odorous products, including benzaldehyde, benzil, acetone, pinacol, and 1-phenyl-2-methyl-1,2-propanediaol. Another photoinitiator fragment identified by Salthammer is 1-hydroxy-cyclohexyl-phenone (HCPK). This compound can serve as a precursor for benzaldehyde, benzil, and cyclohexanone. The chemistry leading to these products is summarized in Salthammer's papers.

### *Building Materials*

Wolkoff[42] has recently summarized the measurement and evaluation of volatile organic compounds from various building materials. In this paper he contrasts "secondary emissions" with "primary emissions". By "secondary", he means emissions of chemicals that were not present in the building material to begin with, but have been formed as a consequence of processes, including chemical transformations, that have occurred after manufacturing. Examples include aldehydes and acids resulting from the ozone- or oxygen-induced oxidation of organic constituents with unsaturated carbon bonds. The ozone/carpet studies discussed earlier[31,32] illustrate this point — a number of compounds were detected in the presence of ozone that were not detected in its absence. Wolkoff concludes, "Emission testing for primary VOC emissions is necessary, but insufficient to characterize the impact of building products in their entire life span on the perceived air quality."

During a 50-day period, Knudsen et al.[43] measured primary and secondary emissions from five common building materials, using small-scale test chambers. Compounds that were tentatively identified as secondary emissions included 2-ethyl-1-hexanol from carpet and PVC floor covering, phenol from PVC floor covering, and several dimethyloctanol isomers from a sealant. The authors suggest that if a building product surface is sensitive to oxidative degradation, increased air velocity may increase secondary emissions. They also state that, in the long term, perceptions of indoor air quality are more likely to be affected by secondary emissions than primary emissions. Odor intensity was used as a measure of indoor air quality.

In a related study, Knudsen et al.[44] examined ozone scavenging by ten different building materials in ventilated test chambers. A sensory panel was used to evaluate emissions from seven of the building materials after they had been exposed to air or air containing a moderate amount of ozone. The ozone levels used in these studies were generally less than 50 ppb. For some of the materials, exposure to ozone affected the panel's perception of the emissions (based on the air quality evaluations). Emissions from certain materials remained modified for some time after the material was exposed to ozone. The authors conclude that, since the study was performed under realistic conditions, the observed effects are likely to occur in actual indoor settings. For this reason, they feel that the ozone content of the air needs to be taken into consideration during emission testing of certain building materials.

Moriske et al.[45] examined the interaction of ozone with wallpaper, latex paint, carpet, plywood, and plaster in test chambers as well as a model home. They found that chemical reactions were a significant sink for ozone, in addition to air exchange and physical decay on surfaces. For most of the materials studied, formaldehyde levels increased markedly in the presence of approximately 70 ppb of ozone. The peak formaldehyde concentration reported was 148 $\mu g/m^3$.

### *Environmental Tobacco Smoke*

The deliberate generation of ozone in indoor settings has been promoted as a method to reduce the concentration of indoor pollutants. Shaughnessy et al.[46] have examined the effect of ozone on the concentrations of VOCs found in environmental tobacco smoke (ETS). Their measurements focused on 18 VOCs, chosen because they represented different types of VOCs and because they were among the most abundant organic constituents of ETS. At moderate ozone concentrations (80 to 115 ppb), ozone had little effect on the concentrations of the monitored VOCs. At high ozone concentrations (1000 to 1400 ppb), ozone reduced the concentration of those compounds with unsaturated carbon–carbon bonds. However, based on a comparison of mass concentrations, this reduction was more than matched by an increase in the concentration of a series of aldehydes, including formaldehyde, acetaldehyde and benzaldehyde. At the lower ozone concentrations, even a very modest ventilation rate (on the order of 0.1 $h^{-1}$) would have produced a greater reduction in the VOCs than that produced by ozone.

## Modeling Indoor Chemistry

### *Hydroxyl Radicals*

Sarwar and coworkers have recently presented results derived from a new indoor air model[47]. The model has been developed using the SAPRC-99 outdoor atmospheric chemistry model and is called the Indoor Chemistry And Particle (ICAP) model. It accounts for transport processes between indoor and outdoor environments, indoor emissions, homogenous chemical reactions, and removal by indoor surfaces. Indoor hydroxyl radical concentrations have been estimated using this new model. The results are basically in agreement with an earlier modeling study[11] and indicate that typical indoor hydroxyl radical concentrations are lower than typical outdoor summertime urban hydroxyl radical levels of 5 to $10 \times 10^6$ molecules/cm$^3$; however, indoor levels can exceed typical nighttime outdoor hydroxyl radical levels of $5 \times 10^4$ molecules/cm$^3$. Effects of selected parameters on indoor hydroxyl radical concentrations have been examined. Indoor hydroxyl radical concentrations increase nonlinearly with increasing outdoor ozone concentrations, indoor alkene emission rates, and air-exchange rates (when outdoor ozone levels are elevated). Indoor temperature and light intensity have a moderate impact on indoor hydroxyl radical concentrations. Outdoor hydroxyl radical concentrations and the removal of hydroxyl radicals by indoor surfaces have a negligible impact on indoor hydroxyl radical concentrations. Production of hydroxyl radicals in indoor environments appears to be controlled primarily by reactions of alkenes with ozone.

## *Effects of Ventilation on Chemistry*

Weschler and Shields[48] used modeling to examine the influence of ventilation on homogeneous chemical reactions among indoor pollutants. Unimolecular and bimolecular reactions occurring indoors were simulated with a one-compartment mass balance approach. The initial modeling assumed steady-state conditions. However, at low air-exchange rates, there may be insufficient time to achieve steady state. Hence, the authors also conducted dynamic modeling of nonsteady-state scenarios. In the cases examined, the results demonstrate that the concentrations of products generated from reactions among indoor pollutants increase as the ventilation rate decreases. This is true for unimolecular and bimolecuar reactions, regardless of whether the pollutants have indoor or outdoor sources. It is also true even when one of the pollutants has an outdoor concentration that displays large diurnal variations. The modeling studies were supplemented with a series of experiments conducted in typical commercial offices. The reaction examined was that between ozone and limonene. The ozone was present as a consequence of outdoor-to-indoor transport, while the limonene originated indoors. Results were obtained for low and high ventilation rates. Consistent with the modeling studies, the concentrations of monitored products were much larger at the lower ventilation rates (even though the ozone concentrations were lower). The results illustrate an additional reason to maintain adequate ventilation in indoor environments — the greater potential at lower ventilation rates for reactions among indoor pollutants to generate reactive and irritating products.

## *Computational Fluid Dynamics*

Sorensen and Weschler[49] have recently applied computational fluid dynamics (CFD) to chemical reactions occurring in an indoor setting. The study examined the reaction of ozone with either d-limonene or α-terpinene (which reacts with ozone about 40 times faster than d-limonene) to produce a hypothetical product. The CFD approach permitted a detailed examination of the influence of imperfect mixing on the ozone/terpene reactions. The scenarios included two air-exchange rates (0.5 and 2.0 $h^{-1}$) and used a floor source for the terpenes with an emission pattern similar to a floor-care product. The chemistry occurred in a fairly large two-dimensional room ($13.6 \times 40.6$ m) with an inlet at the top of the left wall and an outlet at the bottom of the right wall. The room was deliberately scaled so that the Reynolds numbers for key flow regimes matched those of a room in which the calculated flow field had been validated against measured data. For all four scenarios, under steady-state conditions, there were large concentration gradients within the room for the reactants and the hypothetical product. This was due partially to imperfect mixing. However, it also reflected the fact that reactions occurred at different rates across the room (because of varying reactant concentrations) and that the time available for reactions to occur ("age of the air") varied from point to point within the room. The concentrations calculated using the CFD approach, with no mixing assumptions, differed significantly from those calculated using a standard mass-balance approach assuming perfect mixing. The results illustrate that the assumption of perfect mixing, in some cases, will lead to calculated concentrations and exposure estimates that are in considerable error. Further studies are necessary to better define the situations under which such errors will be most significant.

## CONCLUSIONS

The field of indoor chemistry has advanced significantly in the past 5 years. Numerous studies have shown that reactions among commonly occurring indoor pollutants can markedly influence indoor environments. To this author, one of the more important themes running through much of the work is the potential presence of indoor chemically generated species that are not detectable by the analytical methods routinely applied to indoor air. In their mouse bioassay studies, Wolkoff and coworkers have stated, “Addition of the effects of the measured residual reactants and products cannot explain the observed sensory irritation effects. This suggests that one or more strong airways irritants have been formed.” However, the identity of the causative agent(s) remains to be determined. Papers by a number of research groups[20,21,22,23,24] have shown that special derivatization techniques are necessary to identify many of the products of ozone/terpene reactions. Otherwise, these species are unable to pass through standard chromatographic columns. The studies by Glasius et al.[20], together with those from Tobias et al.[18,19], show that special analytical tools are necessary to detect certain products. Such instruments include liquid chromatography/mass spectrometry (LC/MS) and thermal desorption particle beam mass spectrometry. The work of Tobias and colleagues further illustrates that some of the oxidized organic products are thermally unstable. Compounds such as secondary ozonides and selected organic peroxides may exist for a few hours or less at room temperature — long enough to have an effect on human occupants, but too short (and too thermally delicate) for routine analysis. Photoinitiators discussed by Salthammer[41] and used in UV-cured coatings are themselves inherently unstable. The hydroxyl radical[10,11,47], and other radicals expected to be present as a consequence of reactions initiated by the hydroxyl radical, have extremely short lifetimes and can only be detected with special analytical methods. Additional examples of “stealth” indoor pollutants are expected as a consequence of future studies in indoor chemistry. The existence of short-lived, highly reactive compounds, thermally labile compounds, and multifunctional species that are difficult to chromatograph should serve as a warning to indoor air investigators who decree that an indoor setting is free of harmful chemicals. We must be extremely careful not to succumb to a certain hubris regarding our ability to detect irritating or harmful chemicals in indoor settings.

## ACKNOWLEDGMENTS

The author acknowledges coworkers in the cited studies as well as investigators who shared drafts of their work prior to publication. This paper was originally presented as #291 at the Annual Meeting of the Air and Waste Management Association, Orlando, FL, June 2001.
